# Institutional quality and resource-based economic sustainability: the mediation effects of resource governance

**DOI:** 10.1007/s43546-021-00195-x

**Published:** 2022-01-20

**Authors:** Jonathan Mukiza Peter Kansheba, Mutaju Isack Marobhe

**Affiliations:** 1 Department of Management, Universitetet i Agder, Kristiansand, Vest-Agder Norway; 2 Department of Business Studies, Ardhi University, Dar Es Salaam, Tanzania; 3 Department of Finance & Accounting, Tanzania Institute of Accountancy, Dar Es Salaam, Tanzania; 4 SSM-ESAMI Research Centre, Swiss School of Management, Bellinzona, Switzerland

**Keywords:** Resource-based economic sustainability, Resource governance, Institutional quality, Extractive resources, Economic regions

## Abstract

The discovery of extractive resources is associated with multiple opportunities and unbridled optimism on achieving socio-economic development for many countries. However, the question how the host governments meet expectations of indigenous people by ensuring an ideal resource-based economic sustainability (RES) has been receiving less research attention. Using the global panel dataset of 80 resource-endowed economies from 2010 to 2017, we postulate and empirically examine the mediating effect of the resource governance (RESOGV) on the relationship between institutional quality (IQ) and RES. The ANOVA and post hoc ANOVA results revealed significant disparities in terms of IQ, RESOGV, and RES among countries with different levels of economic development, regions, and sector (mining or petroleum). Moreover, the fixed effects (FE) and ordinary least square (OLS) results confirmed that IQ marginally influenced RES. However, the influence was more pronounced when it was partially (but complementarily) mediated by the RESOGV. The paper, therefore, stresses the importance of host governments in improving the quality of their institutions. This will ultimately help enhancing their RESOGV capabilities for attaining economic, social and environmental sustainability.

**Supplementary Information:**

The online version contains supplementary material available at 10.1007/s43546-021-00195-x.

## Introduction

Natural resource endowments in hosting economies are intricately linked with multiple opportunities and unbridled optimism on advent socio-economic development (Van der Ploeg 2011). Extant literature shows that while some economies significantly benefit from their resource endowments, others experience the resource curse (Kirshner and Power 2015). Countries such as Norway and Botswana exemplify the fact that natural resources endowment can be a blessing by fueling economic growth and improving the living standards (Abdo 2014; Stiglitz 2004). On the other hand, resource endowment in other countries has been equated to be a curse resulting into social unrests, poor economic growth, and deteriorating living standards (Norman 2009). In that regard, Ojakorotu and Olaopa (2016) report that more than 50% of Nigerians live in extreme poverty due to widespread corruption and violence relating to the country’s oil extraction. The ideal resource-based economic sustainability can be realized when the hosting economies meet the expectations of indigenous people with the socio-economic benefits derived from the extraction of resources (Apergis and Payne 2014).

Based on the sustainability perspective, the resource-based economic sustainability (RES) refers to an economy characterized by efficient and effective utilization of resources that holistically results into social, economic, and environmental benefits (Waterworth and Bradshaw 2018). It involves designing the policies that help striking the balance between economic growth, social prosperity, and environmental preservation for the multi-generational benefits (Amiri et al. 2019). Developing countries that are endowed with natural resources have been failing to achieve this balance due to over dependence on resource rents that leads to over extraction of exhaustible resources providing a quick boost in economic growth but at the expense of the environment and social welfare (Barbier 2010; Ross 2012). The inability to RES is associated with poor management of natural resources in the form of misappropriation of resource revenues due to weak institutions (Ross 2012; Haber and Menado 2011).

Effective resource governance mechanisms need to be designed and implemented for countries to benefit from their resource endowment (Kaufmann 2013). These include mechanisms by which power and responsibilities relating to natural resources are exercised to ensure effective management of resource revenues and value creation for the betterment of the economy and social livelihoods of the people (Natural Resource Governance Framework 2014). Ineffective resource governance mechanisms are associated with increased poverty in resource-endowed countries, culminating in the institutionalization of the initiatives such as the establishment of Natural Resource Charter (NRC) and the Natural Resource Governance Framework (NRGF) aimed to address the resource governance issues (Robinson et al. 2006). Despite the initiatives, governance challenges still exist especially in resource-endowed developing countries (Cust 2017). The African Progress Panel (2013) reported that between 2010 and 2012, about USD 6.8 billion in oil subsidies were misappropriated in Nigeria, apparently due to poor institutions. Institutions create a broad enabling environment for resource governance mechanisms to work effectively towards proper revenue management and value creation (Robinson et al. 2006).

Kaufmann et al. (2007) define institutions as a set of social factors, rules, beliefs, values, and organizations that jointly motivate regularity in individual and social behavior. They are characterized by rule of law, corruption control, regulatory controls, government effectiveness, political stability, and absence of violence (Cust and Harding 2014). Previous studies (Ahmadov and Guliyev 2016) have largely linked the role of institutional quality to other aspects such as state governance and human capital (Aljarallah2019). There are limited studies assessing institutional quality in the context of resource economy. Few studies available such as Robinson et al. (2006) and Mehlum et al. (2006) established the theoretical foundation on the nexus between the quality of institutions and resource curse, without pinpointing the empirical contribution. Moreover, Rossa and Lootty (2012) and Ross (2012) claimed that there was an insignificant causal-effect linkage between IQ and economic growth of resource-blessed countries. In the current study, we extend the horizons of the theoretical scope. We argue that the contribution of institutional quality (IQ) in steering resource-based economic sustainability becomes much pronounced when interceded by resource governance. As such, the hosting governments need to strengthen and direct their institutions towards ensuring the maximum resource management for them to reap the expected benefits by their people.

Our study seeks to attain mainly two contributions. First, we introduce and theorize on the aspect of resource-based economic sustainability (RES) to the literature on resource economy. Current studies focus on the contribution of natural resource to economic growth (economic aspect) (Xue et al. 2019) or local content (the social aspect) (Bulte et al. 2005) as separate research phenomena. However, there is a need to capture both the theoretical and practical dimensions broadening an understanding of the concept of resource-based economic sustainability relatively holistically (capturing economic, social, and environmental dimensions). Second, our study fills the gap of knowledge on the role of IQ on RES. It postulates and empirically examines the mediation role of RESOGV on the linkage between IQ and RES.

The rest of the paper is organized as follows. “Literature review and hypotheses development” provides an extensive review of extant literature related to the subject matter studied and development of the hypotheses. “Methods” presents the methods employed. “Results and discussions” presents results, discussion, and implications of the findings. “Discussions, implications, and avenues for further research” concludes the paper and recommend for avenues for further research.

## Literature review and hypotheses development

### The resource-based economic sustainability (RES)

Natural resources are regarded as blessing to developed and upper middle-income countries such as Norway, Malaysia, and Botswana (Larsen 2005; Frankel 2010). However, resource endowments are negatively perceived as been a curse to other countries, especially the developing ones such as Bolivia, Nigeria, and Angola (Carbonnier et al. 2011). Ross (2012) defined resource curse as the adverse effects of a country’s natural resource wealth on its economic, social, or political well-being. It is worth noting that exploitation of natural resources can lead to immediate boost in economic growth while it may lead to resources depletion, environmental and social problems where there is an absence of good institutions and proper governance mechanisms (Stevens 2011).

According to Waterworth and Bradshaw (2018), RES refers to efficient and effective utilization of resources with the multidimensional benefits in terms of social, economic, and environment. The concept has been linked with the ability of resource hosting countries to steer their sustainable development through proper exploitation of their natural resources (Van der Ploeg 2011; Kirshner and Power 2015). Resource-endowed countries have the potential to achieve economic development through a boost in exports, job creation and spill-over effects of the natural resource industry into other industries such as manufacturing (Wang 2020). Furthermore, with efficient mobilization of resource rents and control of corruption, resource-endowed countries can significantly improve provision of social services, hence propelling peoples’ living standards (Perez and Claveria 2020). Resource-based economic sustainability is not solely inclined to the need to achieve economic and social development, but also the need for enhancing environmental conservation for the benefit of future generations (Azam and Ahmad 2020).

The ability to create a RES depends on the relative better allocation of resource revenues between consumption and development (Busse and Gröning 2013). Countries like Norway have achieved this by creating a sovereign wealth fund (SWF) that governs the fiscal spending of oil revenues, which is contrary to developing countries that overly rely on resource revenue for fiscal consumption (Cust 2017). Furthermore, resource revenues in developing countries are usually misappropriated for political benefits rather than financing developmental projects such as infrastructure, education, and health care (Kirshner and Power 2015). In this regard, some initiatives have been taken to rectify this problem. For instance, the African Mining Vision (AMV) has stressed a need for ensuring economic and social prosperity through improved transparency and value addition to the mineral resources (Robinson et al. 2006). We, therefore, hypothesize that:

**H1**: *There are significant disparities in terms of RES among the countries with different economic development, regions, and resource sectors (mining or petroleum).*

### The concept of institutional quality (IQ)

Natural resources endowment does not guarantee that a country will realize socio-economic development unless there are good quality institutions in place (Olander 2019). Kaufmann et al. (2007) opine that the quality of institutions can be reflected by states’ corruption control, government effectiveness, political stability, and absence of violence. Other indicators include regulatory controls, the rule of law as well as voice and accountability (Rosa and Looty 2012; Cust and Harding 2014). The quality of institutions is central in determining whether natural resource endowment becomes a blessing or a curse for the host country (Haber and Menado 2011). It allows these countries to take full advantage of their natural resources (Mehlum et al. 2006).

The observation by Easterly and Levine (2003) that resource-endowed economies experience slower growth than those with no resources endowments suggests among other things a problem with their institutions. This can be exemplified by slow growth in resource-endowed countries of Nigeria, Zambia, Sierra Leone, Angola, Saudi Arabia, and Venezuela as opposed to higher growth in South Korea, Taiwan, Hong Kong, and Singapore which are not blessed with resources (Barma et al. 2012). The problem is more prevalent in countries with “point resources” which include oil, minerals and plantations that are in nature geographically concentrated in a narrow area (Isham et al. 2003). The institutional quality in these countries is usually poor, resulting into unparalleled power dynamics and unequal division of surplus (Bulte et al. 2005).

Institutions have a tremendous bearing on the country’s economic development (Mehlum et al. 2006). Moreover, institutions are regarded as a factor of production which directly and indirectly impacts economic development by stimulating technological and capital growth (Hu and Zhang 2010). There have been contradictory findings on the role of IQ on the economic aspects not only in the specific economics’ taxonomies but also in general economies. For instance, Ji et al. (2014) and Xue et al. (2019) depict a negative relationship between institutional quality and economic growth. In natural resource economics, the link between IQ and RES is also inconclusive. While Bulte et al. (2005) and Zhang et al. (2008) argue for direct and positive relationship, Hu and Zhang (2010) and Xue et al. (2019) negate the influence of IQ on the economic growth of resource-endowed countries, arguing that the linkages are not that clear and straight forward. We, therefore, hypothesize that:

**H2**: *There are significant disparities in IQ among countries with different economic development, regions, and the resource sector (mining or petroleum).*

In the following section, we theorize the mediating role of resource governance. Institutional quality is vital for shaping the governance aspects of resources which in turn can stimulate economic development. Institutions characterized by observation of the rule of law and low magnitude of corruption may likely demonstrate efficient utilization of resource rents through proper resource governance mechanisms which in turn propel RES.

### The concept of resource governance (RESOGV) and its mediation role

The extent to which resource revenues can be converted into social and economic development is determined by how well the resource governance mechanisms work (Cust 2017). Resource governance refers to norms and processes which depict the mechanisms by which power and responsibilities relating to natural resources are exercised, decisions are made and how local people participate and benefit from the management of natural resources (Natural Resource Governance Framework 2014).

The first most important aspect in resource governance is revenue management which involves allocation of the resource revenue in the national budget, sub national resource sharing and creation of SWF (Natural Resource Governance Framework 2014). Oil, gas, and minerals have the potential to propel social and economic development due to the tremendous amount of the rents they generate. However, this requires meticulous management of their revenues as they are finite, volatile and can cripple other economic sectors (Barma et al. 2012). Improper management of resource revenues leads to over dependence or the rent-seeking behavior which is detrimental to the economy given the finite and volatile nature of the revenues (Barbier 2010). Effective management of resource revenues involves using them to create SWF and to diversify the economy, making it less dependent on resources (Stiglitz 2004). Norway provides an elaborate example as the country used its oil boom to realize socio-economic development by creating efficient SWF and distribute the wealth to stimulate other sectors as well (Abdo 2014).

The second aspect of resource governance is value creation which incorporates resource licensing arrangements, resource ownership contracts, local impact, and taxation agreements (Robinson et al. 2006). Mehlum et al. (2006) postulate that resource-endowed African countries are facing a resource curse because of poor legislations, taxation and licensing arrangements perpetuated by corrupt leaders that are motivated by personal benefits from their countries’ natural resources. The prevalence of poor institutions at the time of resource discovery may potentially lead to resource curse as incumbent leaders tend to refuse surrendering or compromising their powers due to the vested interest that they have in the resource wealth (Bawumia and Halland 2017).

We assert that the influence of the institutional quality on the resource-based economic sustainability is much vivid via the mediation role of the resource governance. The well-designed and implemented (enforced) resource governance policies are important in achieving RES by shaping investment choices as well as outcomes (Robinson et al. 2006). Good institutions promote effective resource governance while the weak ones create the loopholes for corruption, poor fiscal policies, and poor management of resource revenues (Mu and Hu 2018; Ross 2012). The countries such as Venezuela exemplify this problem since the presence of undemocratic and corrupt institutions has resulted into poor governance of its oil endowment as evidenced by the government’s rent-seeking behavior (Kolstad and Wiig 2009). This created dependence on oil revenues for fiscal spending especially social reforms while crippling other sectors such as agriculture in the process (Mu and Hu 2018). Fluctuations in global oil prices, inefficiency in PDVSA (the state-owned oil corporation) and political tensions in 2016 led the country into high unemployment, hyperinflation, hunger, and widespread violence (Kott 2012).

**Fig. 1:**
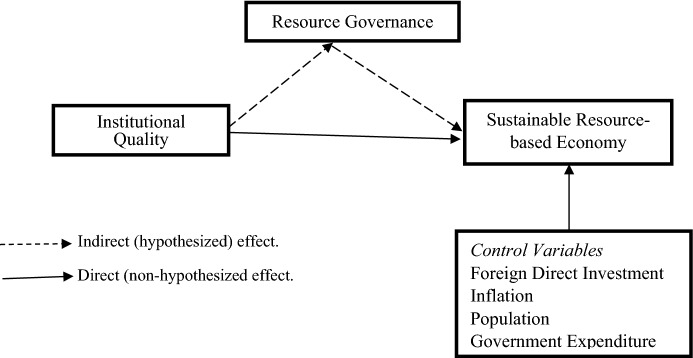
Research model

Resource governance frameworks are designed to ensure that countries realize maximum resource value, effectively manage resource revenues and creating supportive enabling resource investment environment (Dietsche 2017). To achieve RES, countries need to effectively mobilize the resource revenues and build the physical and human capital (Wu 2012), rather than spending on fiscal consumption (Nath 2015). To promote sustainable development from resource endowment, generated revenues can be diversified by investing in other non-resource sectors such as infrastructures and research and development (Nath 2015) (Fig. 1).

**H4**: *There are significant disparities in terms of RESOGV among countries with different economic development, regions, and the resource sectors (mining or petroleum).*

**H5**: *Resource governance (RESOGV) mediates the relationship between the institutional quality (IQ) and the resource-based economic sustainability (RES).*

## Methods

### Data and variable measurement

The study deployed the global dataset from the year 2010–2017 obtained from 80 resource-endowed countries. The dataset was mainly organized from four (4) publicly available global databases, namely World Bank, United Nations Development Program (UNDP), Socio economic Data and Applications Center (SEDAC), and the Natural Resource Governance Institute (NRGI) (see Electronic supplementary material 1).

#### Resource-based economic sustainability (RES)

Our focus was on RES, in which we explored two (2) major subsectors of resource-based economy (mining vis-a-vis oil and gas sectors). Three (3) components were aggregated together to develop a proxy of sustainable resource-based economy; the economic aspect of sustainability measured by GDP per capita growth rate (GDPPG), the social aspect of sustainability measured by Human Development Index (HDI) and the environmental aspect of sustainability measured by Environmental Performance Index(EPI). These three (3) measurements are commonly used to measure any country’s economic sustainability. Thus, we used them in the context of resource-based economies. Shehabi (2021) describes the concept of economic sustainability of resource rich states first focusing on their ability to achieve economic development given the volatility of resource prices (Abdo 2014). The second indicator is the ability to efficiently utilize the resource revenues to boost peoples’ social livelihoods as well as ensuring environment conservation by minimizing pollution and land degradation (Stiglitz 2004). Thus, the RES is an average of the three components (Qiang and Jian 2020) for each year using the following formula:

1 $$RESit = \sum \left( {GDPPGit; \,HDIit;\,EPIit} \right)/3.$$

#### Resource governance (RESOGV)

The study deployed the resource governance index as the proxy for resource governance. The index was recommended by the Natural Resource Governance Institute. It is composed of three (3) subcomponents, namely: value realization, revenue management and enabling environments.

#### Institutional quality (IQ)

The study operationalizes the institutional quality as a proxy computed from six (6) parameters developed by Kaufman et al. (2007) namely: control of corruption, government effectiveness, political stability, and regulatory quality, rule of law and voice and accountability. These were also used by other scholars (Rodrik et al. 2004; Ji et al. 2014) in explaining disparities in the quality of institutions among countries. Thus, the IQ is an average of the six parameters (Kaufman et al. 2007) using the following formula:

2 $$IQit = \sum \left( {CCit + GEit + PSit + RQit + RLit + VAit} \right)/6.$$

#### Control variables

We further included other variables that have an influence on sustainable resource-based economy (SRE) for robust reasons. These variables are lag of RES, lag of IQ, foreign direct investment, population growth, inflation, and government expenditure (Table 1).

**Table 1:** Variable operationalization and data source

Variable	Measurement (s)	Source
Resource-based economic sustainability (RES)	RES is measured as the proxy of the following: -Economic aspect (GDP per capita growth rate) -Social aspect (human development index) -Environmental aspect (environmental performance index)	World Bank UNDP-Human Development Data Center YCELP &CIESIN: NASA (SEDAC). https://doi.org/10.7927/f54c-0r44
Resource governance (RESOGV)	Resource governance index	Natural Resource Governance Institute (NRGI)
Institutional quality (IQ)	IQ is measured as the proxy of the following: -Control of corruption -Government effectiveness -Political stability and absence of violence -Regulatory quality -Rule of law -Voice and accountability	World Bank Governance Indicators (WGI)
Level of economic development	1 if the country is developed economy, 0 otherwise	World Economic Forum
Economic zones	1 = East Asia and Pacific, 2 = Europe and North America, 3 = Latin America, 4 = Middle East and North Africa, 5 = Sub-Saharan Africa	World Economic Forum
Foreign direct investment (FDI)	Net out flow as % of GDP	World Bank
Population (POP)	Population growth rate	World Bank
Inflation (INFL)	GDP deflator (annual %)	World Bank
Government expenditure (GVEXP)	Government expenditure as % of GDP	World Bank

### Model goodness-of-fit and estimation

Different model assumptions were tested (see results in Electronic supplementary material 2, 3 and 4) prior to analyses based on the overall models under panel (FE) and OLS regressions. The Breusch–Pagan test for testing the presence of heteroskedasticity problem (when the error variances are not constant) was performed (Hausman and Taylor 1981). The results show the *p* values under all models were greater than 0.05, indicating the absence of heteroskedasticity problem (Marobhe 2021). The multicollinearity problem was tested using the variance inflation factor (VIF). The VIF results for explanatory variables were less than the cutoff points of 5, suggesting the absence of serious multicollinearity problem (Kansheba 2020). We also tested for normality problem using the Shapiro–Wilk *W* normality test. The results show the *p* values under all models were greater than 0.01, suggesting that residuals are normally distributed (Hair et al. 2010).

Furthermore, the model specification problem was tested. The link test for model specification results shows the *p* values were greater than 0.05, suggesting that the models were correctly specified (Lensink et al. 2018). Statistically significant F-statistics further confirms the goodness-of-fit of the models and that they were correctly specified (Bell et al. 2019). The *R*-squared from the FE results (Table 4) show that the explanatory variables explain up to 12.3% of the variation in the outcome variable. Moreover, the R-squared from the OLS results (Tables 5 and 6) show that explanatory variables explain the variation in the outcome variable by 26.1% in 2013 and 38.4% in 2017.

After ensuring the model goodness-of-fit, we employed the regression models to test the postulated relationships. We first employed a panel 2010–2017 model, in which the fixed effects (FE) estimator was selected over the random effects (RE) estimator following the Hausman test (Agyapong et al. 2019 ). For results robustness and given that the mediating variable comprised the dataset of 2013 and 2017 only, we performed separate OLS regressions for these years. The OLS results enriched our discussion by allowing a specific mediation effect comparison of the RESOGV between these 2 years.

## Results and discussions

### Descriptive statistics and correlation results

Table 2 presents the descriptive statistics of the variables used in the empirical investigation. The output variable, the resource-based economic sustainability exhibits a mean and standard deviation of 39.9% and 11.7%, respectively. The resource governance shows the mean and standard deviation of 12.2% and 22.7%, respectively. The deviations are not close to the mean, which indicates significant disparity among resource-endowed countries in terms of governance of their natural resources for the betterment of their countries. The institutional quality has the mean value of − 0.48 and the standard deviation of 0.76. The dispersion suggests significant variations among resource-endowed developed and developing countries in terms of institutional quality (Polterovich et al. 2010). The correlation matrix and the variance inflation factor results suggest an absence of serious multicollinearity problem. The correlation scores are below the cutoff of 0.7 while the VIF scores are below the threshold of 5.

**Table 2:** Descriptive statistics and correlations

Variable	Obs	Mean	Std. dev	VIF	RES	RESOGV	IQ	FDI	INFL	GVEXP	POP
RES	640	0.399	0.117		1.00						
RESOGV	160	0.122	0.227	2.34	0.36*	1.00					
IQ	640	− 0.484	0.764	2.49	0.44*	0.74*	1.00				
FDI	573	1.094	2.049	1.15	0.09	0.07	0.26*	1.00			
INFL	623	6.313	9.428	1.08	− 0.09	− 0.09	− 0.18*	− 0.08	1.00		
GVEXP	445	21.745	9.097	1.22	0.08	0.25	0.22*	0.03	− 0.07	1.00	
POP	634	2.011	1.310	1.08	− 0.36*	− 0.25*	− 0.14*	0.03	− 0.01	− 0.17*	1.00

*RES* stands for resource-based economic sustainability, *RESOGV* stands for resource governance, *IQ* stands for institutional quality, *FDI* stands for foreign direct investment, *INFL* stands for inflation, *GVEXP* stands for government expenditure, *POP* stands for population growth rate, *VIF* stands for variance inflation factor

### Institutional quality, resource governance and resource-based economic sustainability disparity

Table 3 presents the ANOVA and post hoc ANOVA results aimed to analyze the disparity among resource-endowed economies in terms of resource-based economic sustainability, resource governance and institutional quality. The tests were aimed to test the hypotheses on whether there were significant differences between groups in terms of mentioned variables or not. The results in Table 3 show that there is statistically significant difference in terms of resource-based economic sustainability among resource-endowed economies with different levels of economic development, economic regions and between typologies of the sector (mining and oil and gas sectors), thus, supporting the hypothesis 1. The results partly support the hypotheses 2 and 3, implying there was statistically significant difference of institutional quality and resource governance among the resource-endowed economies with different levels of economic development and economic regions. In addition, there was no significant difference in terms of sector.

**Table 3:** ANOVA and post hoc ANOVA results

	ANOVA					
	Resource-based economic sustainability		Resource governance		Institutional quality	
	*F*-stat	*p* value	*F*-stat	*p* value	*F*-stat	*p* value
Economic develop	107.76	0.000***	28.99	0.000***	276.703	0.000***
Economic regions	63.17	0.000***	8.54	0.000***	23.26	0.000***
Sector	8.73	0.003**	0.37	0.5425	0.34	0.5616

Post hoc ANOVA						
	Resource-based economic sustainability		Resource governance		Institutional quality	
Economic regions	Contrast	*p* value	Contrast	*p* value	Contrast	*p* value
2vs1	0.014	0.865	0.079	0.425	0.172	0.026
3vs1	− 0.018	0.732	0.097	0.205	0.020	0.997
4vs1	− 0.072	0.000***	− 0.072	0.423	− 0.136	0.072
5vs1	− 0.141	0.000***	− 0.078	0.248	− 0.255	0.000***
3vs2	− 0.032	0.187	0.018	0.995	− 0.152	0.068*
4vs2	− 0.087	0.000***	− 0.151	0.005*	− 0.309	0.000***
5vs2	− 0.156	0.000***	− 0.158	0.001**	− 0.428	0.000***
4vs3	− 0.055	0.000***	− 0.169	0.001**	− 0.156	0.025
5vs3	− 0.124	0.000***	− 0.175	0.000***	− 0.275	0.000***
5vs4	− 0.069	0.000***	− 0.006	1.000	− 0.119	0.044**

1 = East Asia and Pacific, 2 = Europe and North America, 3 = Latin America, 4 = Middle East and Northern Africa, and 5 = Sub-Saharan Africa

*, **, and *** denote statistical significance at the 10%, 5%, and 1%, respectively

The post hoc ANOVA helped to reveal which groups were different from the point of view of the categorical variables with more than two levels (groups). In this regard, given that the variable (economic regions) had five (5) groups (East Asia and Pacific, Europe and North America, Latin America, Middle East and North Africa and Sub-Saharan Africa), the post hoc ANOVA test was necessary since it could help depicting how the regions differed from each other. Results in Table 3 reveal statistically significant differences only in some pairs of the economic regions, while others exhibit no difference. In terms of resource-based economic sustainability, Europe and North America outperform all other regions followed by East Asia and Pacific with Sub-Saharan Africa performing the least. In so far as the aspect of resource governance was concerned, Europe and North America and Latin America outperformed all other regions. The results for institution quality suggest that Europe and North America performed better than other regions while Sub-Saharan Africa indicated the poorest quality of institutions.

### The mediation effect of resource governance on the linkage between institutional quality and resource-based economic sustainability

Table 4 presents the fixed effects (FE) result for the mediation of the resource governance on the relationship between institutional quality and resource-based economic sustainability. Model 1 is the baseline, including only control variable. At this stage, foreign direct investment was found to have statistically and positive significant influence on resource-based economic sustainability. Furthermore, we followed the Baron and Kenny (1986) and Zhao et al. (2010) steps of testing the mediation effect. First, we analyzed the influence of the independent variable and mediating variable on the dependent variable separately. Models 2 and 4 were aimed to analyze the influence of the independent variable (the institutional quality) on the dependent variable (resource-based economic sustainability) and the mediating variable (resource governance), respectively. Model 3 was used as the yardstick to analyze the influence of the mediating variable on the dependent variable. In model 5, we analyzed the influence of both variables (independent and mediating) on the dependent variable. This done, we identified the type of mediation by comparing the magnitude and directions of the estimated coefficients revealed in models 2, 3, 4 and 5 (Zhao et al. 2010).

**Table 4:** Fixed effects (FE) results

	*RES*	*RES*	*RES*	*RESOGV*	*RES*
	Model 1	Model 2	Model 3	Model 4	Model 5
	Coef.	Coef.	Coef.	Coef.	Coef.
Lag. resource-based economic sustainability (SRE)	− 0.091 (0.078)	− 0.094 (0.077)	− 0.094 (0.322)		− 0.073 (0.278)
Foreign direct investment	0.053* (0.029)	0.053* (0.028)	0.091 (0.078)	− 0.028 (0.116)	0.115 (0.075)
Inflation	− 0.037 (0.035)	− 0.033 (0.034)	− 0.105* (0.056)	− 0.060 (0.076)	− 0.103* (0.057)
Government expenditure	− 0.044 (0.034)	− 0.054 (0.035)	− 0.107 (0.101)	0.021 (0.068)	− 0.086 (0.086)
Population	0.133 (0.097)	0.110 (0.092)	0.129 (0.177)	0.018 (0.240)	0.111 (0.174)
Lag. institutional quality (IQ)		− 0.220 (0.151)		0.167* (0.087)	− 0.680 (0.494)
Institutional quality (IQ)		0.353* (0.184)		0.113** (0.050)	0.565 (0.427)
Resource governance (RESOGV)			0.122* (0.064)		0.136** (0.055)
_Cons.	0.418*** (0.033)	0.460*** (0.049)	0.371** (0.123)	0.502*** (0.065)	0.323** (0.100)
*R*-squared (within)	0.022	0.045	0.07	0.021	0.123
Chi-squared	2.77**	2.38**	2.24**	2.41**	2.14**
Observations	560	560	160	160	160
No. countries	80	80	80	80	80

In parentheses are robust standard errors

*, **, *** denote statistical significance at 10%, 5%, and 1%, respectively

The results in model 2 show that the institutional quality had weak (at 10%) statistic significant positive effects on the sustainability of resource-based economic. This coincides with our theoretical formulations on the weak influence of institutional quality in fostering resource-based economic sustainability. However, the results in model 4 revealed strong (at 5%) statistical and positive significant effects of institutional quality on the resource governance. Results in model 3 show a weak (at 10%) statistical and positive significant effect of resource governance on resource-based economic sustainability. Model 5 was used to examine the mediation effects of resource governance on the linkage between institutional quality and resource-based economic sustainability. The results confirm the hypothesis 4, showing complementary partial positive statistically significant mediation effects. This suggests that good institutions enhance the governance of resources which ultimately foster resource-based economic sustainability. This can be further signified by comparing the magnitude of the estimated coefficients in models 3 and 5. The results show an increase in the effect of resource governance on resource-based economic sustainability from 0.122 (as a standing-alone predictor in model 3) to 0.136 (as a mediator in model 5). Such an increase is further justified by the disappearance of the significant effect of institutional quality in model 5 unlike in model 2 which suggests its effects is channeled via the resource governance (Zhao et al. 2010).

In addition, separately performed OLS regression results for 2013 (in Table 5) and 2017 (in Table 6) reveal similar findings. Moreover, by comparing the magnitudes of the estimated coefficients in model 10 and 15, respectively, from Tables 5 and 6, it is evidenced that the mediation effect of the RESOGV was higher in 2013 than in 2017 by about 5%.

**Table 5:** OLS results-2013

	*SRE*	*SRE*	*SRE*	*RESOGV*	*SRE*
	Model 6	Model 7	Model 8	Model 9	Model 10
	Coef.	Coef.	Coef.	Coef.	Coef.
Foreign direct investment	0.032 (0.0830)	0.0514 (0.767)	0.015 (0.079)	− 0.024 (0.101)	0.053 (0.077)
Inflation	− 0.009 (0.067)	− 0.0242 (0.060)	− 0.008 (0.064)	0.111 (0.079)	− 0.0175 (0.062)
Government expenditure	− 0.002 (0.065)	− 0.077 (0.0604)	− 0.065 (0.064)	0.114 (0.079)	− 0.084 (0.061)
Population	0.367** (0.113)	0.311** (0.102)	0.292** (0.109)	− 0.206 (0.134)	0.298** (0.104)
Institutional quality (IQ)		0.128* (0.066)		0 .280*** (0.038)	0.112 (0.075)
Resource governance (RESOGV)			0.228** (0.071)		0.251** (0.127)
_Cons	0.455*** (0.033)	0.503*** (0.032)	0.349*** (0.045)	0.568*** (0.042)	0.468*** (0.059)
Adj *R*-squared	0.086	0.266	0.187	0.500	0.261
F-statistics	2.85**	6.74***	4.63**	16.54***	5.65**
Observations	80	80	80	80	80
No. countries	80	80	80	80	80

In parentheses are robust standard errors

*, **, *** denote statistical significance at 10%, 5%, and 1%, respectively

**Table 6:** OLS results-2017

	*SRE*	*SRE*	*SRE*	*RESOGV*	*SRE*
	Model 11	Model 12	Model 13	Model 14	Model 15
	Coef.	Coef.	Coef.	Coef.	Coef.
Foreign direct investment	0.085 (0.114)	0.154 (0.115)	0.010 (0.116)	− 0.078 (0.117)	0.161 (0.116)
Inflation	− 0.045 (0.068)	− 0.028 (0.063)	− 0.0105 (0.067)	0.000 (0.063)	− 0.028 (0.063)
Government expenditure	0.005 (0.058)	− 0.067 (0.055)	− 0.045 (0.059)	0 .074 (0.055)	− 0.0607 (0.055)
Population	0.627*** (0.128)	0.592*** (0.115)	0.616*** (0.124)	0 .027*** (0.115)	0.589*** (0.115)
Institutional quality (IQ)		0.137* (0.071)		0.299*** (0.031)	0.127 (0.096)
Resource governance (RESOGV)			0.214** (0.083)		0.238** (0.119)
_Cons	0.466*** (0.031)	0.514*** (0.029)	0.373*** (0.046)	0.536*** (0.029)	0 .559*** (0.069)
Adj *R*-squared	0.239	0.387	0.337	0.646	0.384
F-statistics	7.19**	11.01***	7.51***	29.85***	9.21***
Observations	80	80	80	80	80
No. countries	80	80	80	80	80

In parentheses are robust standard errors

*, **, *** denote statistical significance at 10%, 5%, and 1%, respectively

**Fig. 2:**
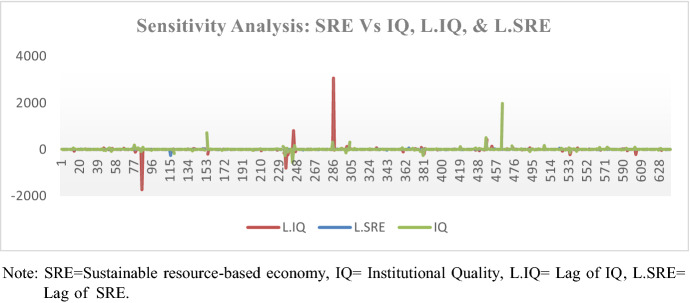
Sensitivity analysis. *SRE* sustainable resource-based economy, *IQ* institutional quality, *L.IQ* lag of IQ, *L.SRE* lag of SRE

We further performed the sensitivity analysis to examine how the dependent variable (the resource-based economic sustainability) was vulnerable to changes in its predictors. However, the resource governance was not included due to data limitation (missing the data during some periods). The analysis involved mainly three variables: institutional quality, lag of institutional quality as well as lag of resource-based economic sustainability. It was necessary to include the lag variables given that they are theoretically believed to have an influence on the output variable. The results in Fig. 2 show that the resource-based economic sustainability is not much sensitive to the mentioned variables. The percentage changes of the output variables to independent variables seemed to be fairly stable. This further coincides to our theorizing that the effect of institutional quality to the resource-based economic sustainability is much pronounced when mediated by resource governance.

## Discussions, implications, and avenues for further research

### Discussion

The study sought to examine the impact of institutional quality in resource-endowed countries. The underlined motivation behind the study was to comprehend how ideal resource-based economic sustainability could be attained in the said countries. The study has established that the impact of institutional quality becomes more vivid when mediated by resource governance. Our examination focuses on the petroleum (oil and/or gas) and mineral sectors in different regions across the globe. First and foremost, our study assessed disparities among countries from different regions, with different economic development levels and across the sector type in terms of institutional quality, resource governance and resource-based economic sustainability. Our findings indicated significant disparities among regions in all three (3) examined aspects. North America and Europe region appeared to perform better in terms of institutional quality, resource governance and resource-based economic sustainability while Sub-Sahara Africa performed poorly in all three (3) aspects. These findings relate to our other observation which showed significant differences between the three (3) aspects with respect to economic development levels.

Resource-endowed countries in North America and Europe are either high-income or upper middle-income countries (The World Bank 2019). These countries are characterized by effective institutions which have a bearing on their economic success (Acemoglu et al. 2014). The results help explaining the poor performance exhibited by Sub-Sahara Africa in all three (3) aspects as most of the resource-endowed countries in this region are low-income countries with a few lower middle-income ones (The World Bank 2019). The poor conditions in these countries are explained by the presence of weak and corrupt institutions, leading to uneven distribution of the resource wealth (Norman 2009; Ojakorotu and Olaopa 2016).

Disparities in resource governance are explained using examples from developed European countries such as Norway which exemplifies how resource governance mechanisms could function. The country has been able to do this by the creating a SWF managed by highly skilled professionals (Cust 2017). During the period from 1999 to 2010, the country had invested USD 560 million out of USD 1 trillion oil revenues in its SWF which helped the country to escape the rent-seeking behavior, thus reducing over dependence on oil revenues (Kott 2012). This also helped preventing appreciation of the country’s currency, hence ensuring survival and growth of its other export led economic sectors such as fishing (Abdo 2014).The SWF also contributed towards protecting the country’s economy during the periods of deterioration of the oil prices (Kott 2012). This is similar to Qatar, which borrowed USD 7.6 billion from its USD 300 SWF during the economic down-turn caused by COVID-19 to finance the budget deficit (National Resource Governance Institute 2020).

Our findings have shown poor resource governance in other regions such as Sub-Sahara Africa. The resource-endowed countries in this region such as Nigeria, Gabon, Angola, and South Sudan inefficiently used their resource booms to fuel the public expenditure, resulting into a huge budget imbalance and skyrocketing public debts (Natural Resource Governance Institute 2019). The Governments in this region do not have in place proactive measures to adhere to the very rules and regulations set to govern management of resources assets, the shortcoming that has resulted into misappropriation (Ayee 2014). The lack of proactive measures of natural resources governance in these countries has led to gross mismanagement of natural resources propelling corruption and overdependence on resource revenues (Aluu 2019).

Our results indicate a weak influence of institutional quality on achieving a resource-based economic sustainability. This is consistent with the previous findings (Olander 2019) and our theoretical reflection on a “no (OR weak)-direct impact” of institutional quality on resource-based economic sustainability. However, we support the positive mediation (but complementary) effect of resource governance on the aforementioned relationship. Our findings suggest that good institutions at the time of resource discovery set a sound and firm foundation for resource governance mechanisms to operate, which in turn stimulates eventual socio-economic development (Ross 2012). A resource-endowed country can experience a resource curse when it has poor capabilities to properly manage the resource revenues caused by low-level institutions (Mu and Hu 2018).

These results are partly different from those of Qiang and Jian (2020) and Xue et al. (2019) that postulate a direct significant relationship between institutional quality and economic growth. The variations in findings can be attributed to the fact that previous studies focused on a single aspect of sustainability (economic growth) while neglecting the social and environmental aspects. Though economic growth is a positive indicator of development, it is not sufficient to portray the entire socio-economic development picture. By the way, there are countries that have high economic growth but exhibit extremely poor social development and/or environmental concern (Bulte et al. 2005).

### Implications and avenues for further research

This study has theoretical and practical contribution based on two (2) premises. First, the study has integrated the concepts of resource governance, institutional quality, and resource-based economic sustainability. Extant research (e.g., Apergis 2014; Aluu 2019; Aljarallah 2019) have tended to explore the concept of resource endowments and resulted resource curse with a limited focus on the causal relationships between the aforementioned phenomena. This study has shed some light on the holistic and integrative theoretical model framing the role of resource governance and institutional quality on ensuring a wide spectrum of the resource sector benefits in terms of economic, social, and environmental contribution. Second, the study offers empirical evidence that may be beneficial to scholars, policy makers and practitioners in carrying out future research or formulating the policies aimed to ensuring efficient exploitation of natural resources.

Furthermore, the disparity in terms of resource governance, institutional quality as well as resource-based economic sustainability among countries with different economic development levels and regions herald the need for further inquiry. The study provides an important lesson to resource-endowed developing economies to revisit their policies on the resource sector paying close attention to institutional quality as a foundation for effective resource governance. Our findings suggest that resource-endowed developed economies provide exemplary benchmarks to those developing. We further argue that developing countries should consider their contextual settings and needs while utilizing resource governance and institutional models applied by developed economies.

The study further advocates for resource-endowed countries especially developing ones to recognize the importance of building good institutions to reap the benefits of their resource endowments. Previous research (Nath 2015) has shown that most developing countries are facing the resource curse syndrome. However, countries like Botswana have managed to avoid the resource curse and realize sustainable economic growth despite that the country is one of the developing countries. This provides a fertile ground for advancing research and scholarship with a view to providing a scholarly explanation of the paradox. In that regard, further studies are needed to investigate how Botswana has been able to turn their resources into a blessing irrespective of the fact that the country is locate in an entirely different economic zone as opposed to Norway, Australia, Canada, and USA.

## Conclusions

Natural resource endowment provides an opportunity for a country to leapfrog to higher socio-economic development. However, the formulation is contingent to the quality of the country’s institutions upon discovery of resources. Resource endowments in developing countries such as those in Sub-Saharan Africa have turned out to be a curse attributed to poor institutions characterized by widespread corruption and undemocratic government processes. These have led to lack of transparency in resource exploitation contracts and inefficient revenue management with detrimental effects on promoting the resource- based economic sustainability. The poor governance mechanisms resulting from weak institutions have deepened and entrenched the resource dependence problem. Eventually, a country is subjected to the risk of economic down-turn when prices of resources plummet in the world market. The resource dependence problem causes other economic sectors to crumble in a phenomenon dubbed as the “Dutch disease” which is also prevalent among emerging and developed countries such as Saudi Arabia, Kuwait, and Venezuela.

The findings of our study cement the importance of having good institutions for reaping the benefits entailed in natural resources endowment through enhanced resource governance systems. Good institutions provide a foundation upon which effective resource governance mechanisms are developed, ultimately enabling the country to not only achieve economic growth but also improve the livelihoods of its people as well as preserving the environment. Hence, ensuring economic sustainability should be at the core of the resource-endowed countries’ development agenda for the betterment of the future generations. This can be realized through the establishment of strong, effective and efficient institutions capable of preventing the few greedy individuals entrusted with natural resources stewardship to enrich themselves through the public coffers.

## Supplementary Information

Below is the link to the electronic supplementary material.

Supplementary file1 (DOCX 20 KB)

## Data Availability

The data that support the findings was retrieved from four (4) global databases, namely World Bank, United Nations Development Program (UNDP), Socio economic Data and Applications Center (SEDAC), and the Natural Resource Governance Institute (NRGI).
