# Supplementary material for: Institutional quality and resource-based economic sustainability: the mediation effects of resource governance
Source: SN Bus Econ. 2022 Jan 20;2(2):19. doi: 10.1007/s43546-021-00195-x (PMC8771607; doi:10.1007/s43546-021-00195-x)
Supplement: Supplementary file 1 — Supplementary file1 (DOCX 20 KB) [file 43546_2021_195_MOESM1_ESM.docx]

**Electronic Supplementary Material**

**Appendix 1: Data sources**

| S/N | Variable | Source | Data Link |
| --- | --- | --- | --- |
| 1. | Human development index (HDI) | UNDP | http://hdr.undp.org/en/data |
| 2. | Environmental performance index | SEDAC | https://sedac.ciesin.columbia.edu/data/set/epi-environmental-performance-index-2020 |
| 3. | GDP per capita growth rate (GDPGR) | UN | https://data.worldbank.org/indicator/NY.GDP.PCAP.KD.ZG |
| 4. | Resource Governance (RG) | NRGI | https://resourcegovernanceindex.org/data/both/issue?region=global&years=2017%2C2021 |
| 5 | Institutional Quality (IQ) | World Bank | https://info.worldbank.org/governance/wgi/ |

**Appendix 2: Regression model assumptions (Panel-overall model)**

| **S/N** | **Regression Assumptions** | | | **Test(s)** | | | **We seek values** | |
| --- | --- | --- | --- | --- | --- | --- | --- | --- |
|  |  |  |  | *Breusch-Pagan hettest* | | |  | |
| 1 | No heteroskedasticity problem | | | Chi2(1): 13.076 | |  | | > 0.05 |
|  |  |  |  | p-value: 0.052 | |  | |  |
| 2 | No multicollinearity problem | | | *VIF (See Table.2)* | | | < 5.00 | |
| 3 | Residuals are normally distributed | | | Shapiro-Wilk W normality test  z: 1.030 |  |  | | > 0.01 |
|  |  |  |  | p-value: 0.034 | |  | |  |
|  |  |  |  | *Linktest* | |  | |  |
| 4 | No specification problem | | | t: -1.482 | |  | | > 0.05 |
|  |  |  |  | p-value: 0.140 | |  | |  |
|  |  |  |  | *Test for appropriate functional form* | | | | |
| 5 | No functional form problem | | | F(3,150): 1.476 | |  | | >0.05 |
|  |  |  |  | p-value: 0.223 | |  | |  |
| 6 | No influential observations | | | *Cook's distance* | | | < 1.00 | |
|  |  |  |  | no distance is above the cut-off | | | | |

**Appendix 3: Regression model assumptions (OLS-overall model-2013)**

| **Regression assumptions:** | **Test:** | |  | **We seek values** |
| --- | --- | --- | --- | --- |
| No heteroskedasticity problem | *Breusch-Pagan hettest* | |  |  |
|  | Chi2(1): 3.458 | |  | > 0.05 |
|  | p-value: 0.063 | |  |  |
| no multicollinearity problem | *Variance inflation factor* | |  |  |
|  | resogv : 2.12 | pop : 1.17 |  | < 5.00 |
|  | iq : 2.10 | infl : 1.16 |  |  |
|  | gvexp : 1.28 | fdi : 1.14 |  |  |
| residuals are normally distributed | *Shapiro-Wilk W normality test* | | | > 0.01 |
|  | z: 2.030 |  |  |  |
|  | p-value: 0.021 | |  |  |
| no specification problem | *Linktest* |  |  | > 0.05 |
|  | t: -1.360 |  |  |  |
|  | p-value: 0.178 | |  |  |
| appropriate functional form | *Test for appropriate functional form* | | | > 0.05 |
|  | F(3,70):1.578 | |  |  |
|  | p-value: 0.202 | |  |  |
| no influential observations | *Cook's distance* | |  | < 1.00 |
|  | no distance is above the cutoff | | |  |

**Appendix 4: Regression model assumptions (OLS-overall model-2017)**

| **Regression assumptions:** | **Test:** | |  | **We seek values** |
| --- | --- | --- | --- | --- |
| No heteroskedasticity problem | *Breusch-Pagan hettest* | |  |  |
|  | Chi2(1): 5.958 | |  | > 0.05 |
|  | p-value: 0.052 | |  |  |
| no multicollinearity problem | *Variance inflation factor* | |  |  |
|  | resogv :3.02 | pop : 1.07 |  | < 5.00 |
|  | iq : 3.54 | infl : 1.12 |  |  |
|  | gvexp : 1.20 | fdi : 1.34 |  |  |
| residuals are normally distributed | *Shapiro-Wilk W normality test* | | | > 0.01 |
|  | z: 3.530 |  |  |  |
|  | p-value: 0.041 | |  |  |
| no specification problem | *Linktest* |  |  | > 0.05 |
|  | t: -2.589 |  |  |  |
|  | p-value: 0.012 | |  |  |
| appropriate functional form | *Test for appropriate functional form* | | | > 0.05 |
|  | F(3,70):2.990 | |  |  |
|  | p-value: 0.371 | |  |  |
| no influential observations | *Cook's distance* | |  | < 1.00 |
|  | no distance is above the cutoff | | |  |
